# ADAR1: a central regulator of dsRNA sensing in host-virus interactions

**DOI:** 10.3389/fimmu.2026.1756517

**Published:** 2026-02-10

**Authors:** Congnuan Liu, Younho Choi

**Affiliations:** Florida Research and Innovation Center, Cleveland Clinic, Port St. Lucie, FL, United States

**Keywords:** ADAR1, A-to-I editing, host-virus interaction, PKR, RNA sensing pathways

## Abstract

Adenosine deaminase acting on RNA 1 (ADAR1) is a key regulator of RNA homeostasis and innate immunity through its adenosine-to-inosine (A-to-I) editing of double-stranded RNAs (dsRNAs). By editing endogenous dsRNAs, ADAR1 prevents inappropriate activation of RNA sensors such as PKR, RIG-I, and MDA5, thereby maintaining immune tolerance to self RNA. However, growing evidence indicates that this essential immunomodulatory function of ADAR1 can be exploited by viruses to facilitate infection. Many viruses leverage ADAR1 to suppress RLR- and PKR-mediated signaling, dampen type I interferon responses, and promote viral replication-highlighting a prominent proviral role for ADAR1. Conversely, ADAR1 can also exert antiviral effects, including hyper-editing of viral genomes, disruption of viral RNA structures, and modulation of host antiviral signaling pathways. Thus, ADAR1 acts as a context-dependent regulator of virus-host interactions, functioning both as a guardian against aberrant immune activation and as a host factor co-opted by viruses to establish productive infection. Understanding how viruses manipulate ADAR1 and how ADAR1 differentially impacts PKR and RIG-I/MDA5 pathways will advance our knowledge of viral immune evasion mechanisms and may inform new therapeutic strategies. This review summarizes current insights into the antiviral and proviral roles of ADAR1 during viral infection, with emphasis on viral strategies that finetune ADAR1 activity to shape infection outcomes.

## 1 Introduction

Rapid and accurate detection of invading viruses is fundamental to mounting an effective innate immune response, the first line of antiviral defense. Over more than a billion years of coevolution, hosts have developed sophisticated mechanisms to discriminate self from non-self nucleic acids (1). Viral molecules absent in uninfected host cells-such as genomic DNA, single-stranded RNA (ssRNA), double-stranded RNAs (dsRNAs), RNA with 5’-triphosphate ends, and viral proteins-serve as pathogen-associated molecular patterns (PAMPs) that are recognized by pattern-recognition receptors (PRRs) (1–3). Three major classes of PRRs mediate host surveillance of viral infection: Toll-like receptors (TLRs), retinoic acid-inducible gene I (RIG-I)-like receptors (RLRs), and nucleotide-binding oligomerization domain (NOD)-like receptors (NLRs) (1). TLRs detect viral PAMPs at the cell surface and within endosomes (1), whereas RLRs and NLRs operate as cytosolic sensors of infection (4, 5). Activation of TLRs and RLRs induces type I interferons (IFNs) production (1, 5, 6), while NLRs-particularly inflammasomes-promote the maturation and release of interleukin-1β (IL-1β) and other inflammatory cytokines (1, 7). Collectively, these pathways orchestrate two central antiviral programs: type I IFN-mediated antiviral signaling and IL-1β-mediated proinflammatory responses, enabling robust control of viral infection (8). In addition to these canonical PRRs, protein kinase R (PKR), serving as a critical cytosolic sensor of dsRNA, is also a central component of the innate antiviral defense. Upon binding dsRNA, PKR becomes activated through autophosphorylation and suppresses protein synthesis via phosphorylation of eukaryotic initiation factor 2α (eIF2α), thereby limiting viral replication (9). However, excessive or inappropriate activation of PRR or PKR signaling can trigger harmful outcomes-including cytokine storms, autoinflammation, and autoimmune disease. Thus, host cell must employ regulatory mechanisms to ensure precise detection of viral dsRNAs while avoiding aberrant responses to endogenous dsRNAs (10). Among these regulatory systems, adenosine-to-inosine (A-to-I) RNA editing, mediated by the adenosine deaminase acting on RNA (ADAR) family of enzymes, plays a central role in distinguishing self from non-self dsRNAs (11).

In mammals, three ADAR family members have been identified: ADAR1 and ADAR2, which are catalytically active, and ADAR3, which lacks deaminase activity (11, 12). ADAR1 is the predominant enzyme responsible for A-to-I editing of endogenous dsRNAs, thereby masking self-dsRNA from detection by cytosolic antiviral sensors and preventing inappropriate immune activation (12). Loss-of-function mutations in ADAR1 disrupt this gating mechanism and lead to severe type I interferonopathies and autoinflammatory disorders-including Aicardi-Goutières syndrome (AGS), Dyschromatosis symmetrica hereditaria (DSH), bilateral striatal necrosis (BSN), and spastic paraplegias (13, 14).

Beyond its role in maintaining immune homeostasis, ADAR1 is implicated in diverse biological processes. Dysregulated RNA editing is associated with tumor proliferation, migration, and immune evasion across numerous cancers-including breast, gastric, lung, liver and colon cancers (12). Moreover, ADAR1 plays a multifaceted and context-dependent role in viral infection, exerting both proviral and antiviral effects through editing-dependent and editing-independent mechanisms. By marking self-dsRNAs while allowing detection of pathogenic dsRNAs, ADAR1 fine-tunes PRR signaling and calibrates type I IFN responses to optimize host defense.

Recent studies further indicate that ADAR1 influences viral pathogenesis, viral evolution and host range adaptation-as recently exemplified in emerging viral infections such as SARS-CoV-2 (15). In some contexts, ADAR1 promotes viral replication by suppressing PKR-mediated antiviral signaling, while in others it restricts infection by limiting viral manipulation of host stress pathways. These virus-specific outcomes highlight ADAR1 as a central regulatory node in host-virus interactions, and a promising therapeutic target in infectious disease. In this review, we summarize current insights into the mechanisms by which ADAR1 regulates innate immune sensing and dsRNA editing and discuss how viruses exploit-or are restricted by-ADAR1 activity during infection.

## 2 ADAR1-mediated A-to-I RNA editing

RNA processing mechanisms-including alternative splicing, alternative polyadenylation and diverse RNA modifications-evolved to shape transcriptomic complexity and enhance cellular functionality in higher eukaryotes (16). Nearly all RNA species, including transfer RNA (tRNA), ribosomal (rRNA), messenger (mRNA), and both short and long noncoding RNAs (sncRNA and lncRNA), undergo post-transcriptional modifications that influence RNA-protein interactions and regulate essential aspects of RNA metabolism such as structure, stability, splicing, polyadenylation, localization, transport and translation (17). Dysregulation of RNA modification pathways has been linked to cancer, immune disorders, and neuromuscular diseases (17).

Among more than 100 known RNA modifications, RNA editing is unique because it changes the nucleotide sequence of RNA transcripts relative to the genome (16). Two major classes exist: cytosine-to-uridine (C-to-U) editing, catalyzed by APOBECs (apolipoprotein B mRNA editing catalytic polypeptide-like family), and adenosine-to-inosine (A-to-I) editing, mediated by ADARs on dsRNAs or ADATs (adenosine deaminases acting on tRNAs) on tRNA (18). ADAR was first identified as an RNA unwindase in 1987, followed by the discovery of its dsRNA editing activity in 1988 (19–23). Subsequently, numerous studies demonstrate that ADAR-mediated A-to-I editing on dsRNAs is the most prevalent and mainly occurs in non-coding regions of mRNA. Most editing sites lie within inverted Alu repeats that generate dsRNA structures serving as ADAR substrates. These editing events are essential for cell survival and have been implicated in stress responses, immune regulation, cancer progression, and stem cell fate determination (15, 16, 24, 25).

**Figure 1:**
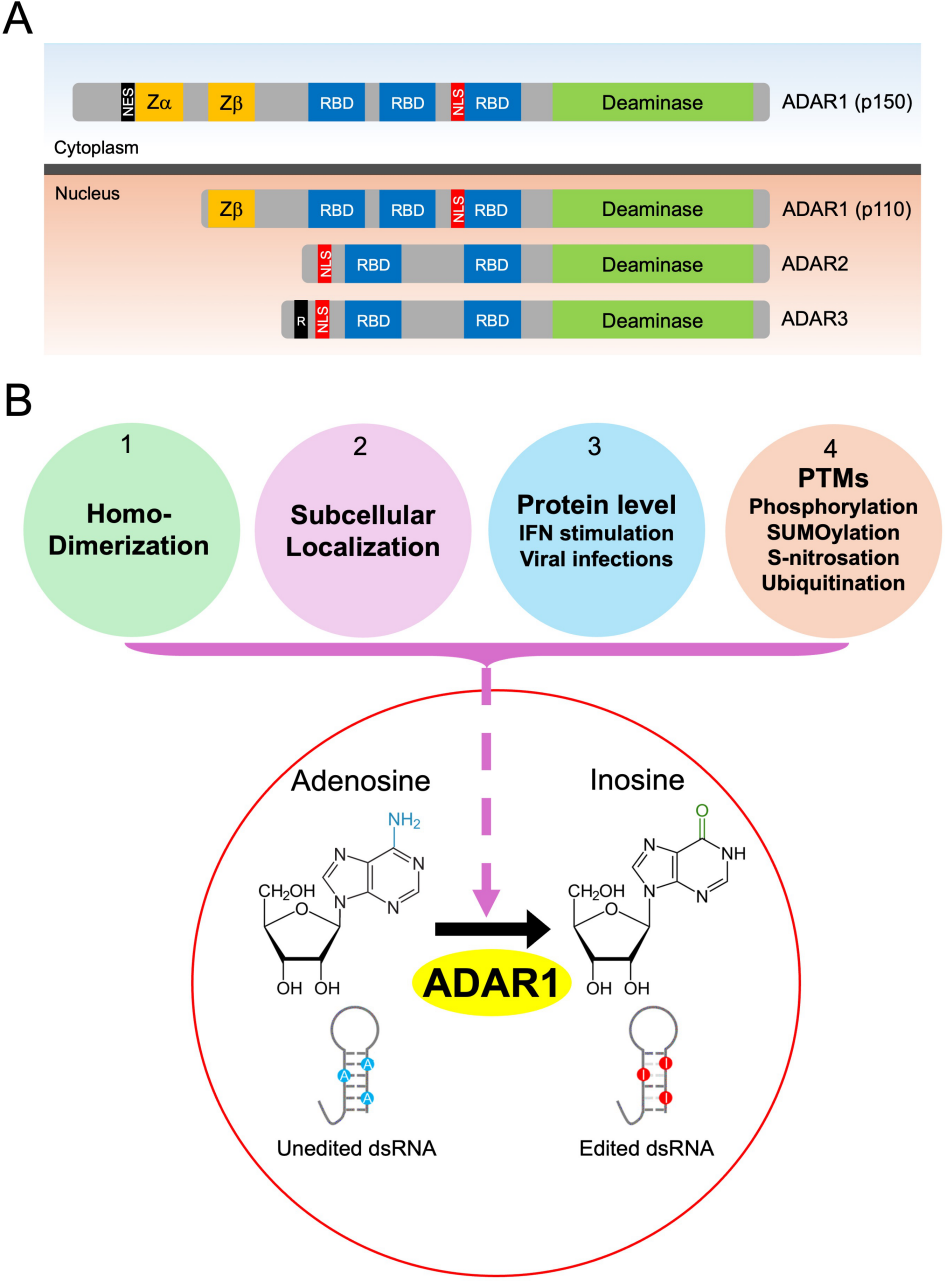
Structure, function, and regulation of ADAR1-mediated A-to-I editing. **(A)** The ADARs family consists of three members: ADAR1, ADAR2, and ADAR3. ADAR1 is expressed as two isoforms, the interferon (IFN)-inducible ADAR1p150 and the constitutively expressed ADAR1p110. ADAR1p150 predominantly localizes to the cytoplasm, whereas ADAR1p110, ADAR2, ADAR3 are primarily nuclear. All ADAR proteins contain double-stranded RNA-binding domains (RBDs), a C-terminal deaminase domain, and nuclear localization signal (NLS). In contrast, N-terminal Z-DNA/RNA-binding domains (Zα and Zβ) are unique to ADAR1. The ADAR1p110 isoform contains only the Zβ domain, whereas ADAR1p150 isoform harbors both Zα and Zβ domains and additionally possesses an N-terminal nuclear export signal (NES) that promotes cytoplasmic localization. ADAR3 has a unique R-domain (R) consisting of a series of arginine residues that are required for binding to single-stranded RNA. **(B)** ADAR1 functions as an RNA editor by catalyzing the conversion of adenosine (A) to inosine (I) within dsRNAs, thereby marking endogenous dsRNAs as “self”. ADAR1-mediated editing is regulated by multiple mechanisms: (1) homodimerization, which is required for catalytic activity; (2) subcellular localization and nucleo-cytoplasmic shuttling, which influence access to RNA substrates; (3) protein abundance, which is modulated by IFN signaling and viral infections; And (4) post-translational modifications (PTMs), including phosphorylation, SUMOylation, S-nitrosation, and ubiquitination, which find-tune ADAR1 stability and enzymatic activity.

The mammalian ADAR family consists of three members: ADAR1, encoded by the ADAR gene; ADAR2, encoded by ADARB1; and ADAR3, encoded by ADARB2 (**Figure 1A**) (26). ADAR1 is broadly expressed across tissues, whereas ADAR2 is predominantly expressed in the brain and ADAR3 is largely restricted to neuronal tissues (16, 25, 27–29). All ADAR proteins share a conserved modular architecture that includes one or more dsRBDs and a C-terminal deaminase domain, which together mediate the conversion of adenosine to inosine at the C6 position within dsRNAs (26). Structurally, ADAR1 is unique in harboring N-terminal Z-DNA/RNA–binding domains (Zα and Zβ) in addition to multiple dsRBDs and the conserved deaminase domain, a feature not shared by ADAR2 or ADAR3 (25, 26, 30, 31).

Functionally, ADAR1 and ADAR2 are catalytically active, with ADAR2 primarily mediating site-specific RNA editing in the central nervous system, whereas ADAR3 lacks detectable deaminase activity and is thought to function mainly as an RNA-binding protein (26, 32, 33). This review primarily focuses on ADAR1, which accounts for most RNA editing events in humans (16, 34). ADAR1 is expressed as two isoforms generated from distinct promoters: the IFN-inducible ADAR1p150 and the constitutively expressed ADAR1p110 (**Figure 1A**). ADAR1p150 is predominantly cytoplasmic, owing to a nuclear export signal located in its N-terminal region near the Zα domain, whereas ADAR1p110 lacks the Zα domain and localizes primarily to the nucleus (25, 35, 36). As a result, ADAR1p150 plays a predominant role in regulating cytosolic RNA-sensing pathways, including suppression of PKR activation by endogenous dsRNAs. Consistent with these functional differences, ADAR1p150 is enriched in immune-related tissues such as the thymus and spleen, while ADAR1p110 is highly expressed in the brain (12, 37). A core function of ADAR1 is the A-to-I editing of endogenous dsRNAs to mark them as “self”, thereby suppressing aberrant activation of cytosolic RNA-sensing pathways mediated by MDA5 (38). Specifically, the cytoplasmic ADAR1p150 isoform uniquely suppresses the MDA5-MAVS signaling axis to prevent an inappropriate IFN response (39). Consistently, ADAR1 mutations associated with AGS more severely impair the editing activity of the cytoplasmic ADAR1p150 isoform than that of the nuclear ADAR1p110 isoform, underscoring the critical role of ADAR1p150-mediated A-to-I editing in preventing cytosolic dsRNA sensing of endogenous transcripts (40). Collectively, this editing activity is essential for maintaining immune tolerance to self dsRNAs and for preventing excessive IFN induction, inflammation, and autoimmunity (12).

### 2.1 Mechanism and regulation of ADAR1 activity

ADAR1-mediated RNA editing begins when the enzyme recognizes and binds to dsRNA duplexes formed by transcripts primarily containing two Alu elements positioned in close proximity but in opposite orientations (12, 16). Upon binding, the catalytic domain of ADAR1 flips the target adenosine out of the double-stranded helix and performs hydrolytic deamination, converting A-to-I within dsRNAs (**Figure 1B**) (34). During translation, inosine is interpreted as guanosine (G) and pairs with cytosine (C); thus, editing sites are detected as A-to-G mismatches in sequencing analyses (16, 25). These A-to-I conversions are essential for distinguishing self-derived dsRNAs from viral dsRNAs and preventing inappropriate activation of host dsRNA sensors.

To ensure the accurate and efficient editing of self-derived dsRNAs, ADAR1 deaminase activity is tightly regulated by multiple mechanisms (**Figure 1B**). Early studies established that ADAR1 homodimerization is a prerequisite for its RNA editing activity (41, 42). More recent work further clarifies the importance of dimer formation, showing that disruption of ADAR1 homodimerization leads to loss of editing in a site-specific manner, suggesting that dimerization may function as a selection mechanism for editing sites (34).

In addition to dimerization, post-translational modifications (PTMs) play a critical role in regulating ADAR1 activity. SUMOylation of ADAR1 at lysine 418 decreases its editing ability without affecting subcellular localization (43). IFN signaling also induces Lys48-linked ubiquitination at lysines 574 and 576 of ADAR1p110, mediated by the E3 ligase β transducing repeat-containing protein, resulting in proteasomal degradation of ADAR1p110 and reduced A-to-I editing (44). Phosphorylation negatively regulates ADAR1 activity as well; phosphorylation of ADAR1p110 at T738 by AKT inhibits its deaminase function (45). More recently, S-nitrosation has emerged as an additional regulatory PTM. Nuclear endothelial nitric oxide synthase promotes S-nitrosation of ADAR1, enabling ADAR1 to suppress dsRNA accumulation and type I IFN signaling, thereby contributing to vascular homeostasis (46).

Subcellular localization is another key determinant of ADAR1 function. Efficient RNA editing is primarily mediated by the cytoplasm-localized ADAR1p150 isoform, which exhibits substantially higher catalytic activity than the nuclear ADAR1p110 (>70-fold) (47). When restricted to the nucleus, ADAR1 cannot access cytosolic RNA substrates and therefore fails to edit many target sequences (48, 49). In addition to localization, ADAR1 expression levels can influence its catalytic activity; however, the relationship between protein abundance and editing frequency is only moderate-and in some contexts weak-indicating that factors beyond expression level critically regulate ADAR1-mediated RNA editing (50, 51).

Collectively, these studies demonstrate that ADAR1-mediated A-to-I RNA editing is governed by a highly coordinated and multilayered regulatory system involving dimerization, PTMs, localization, and expression dynamics (**Figure 1B**).

### 2.2 ADAR1 prevents self-dsRNA from PKR/RLR detection and maintains immune homeostasis

Detection of dsRNAs is a central mechanism of innate immune defense across diverse organisms (52). ADAR1 plays a critical role in this process by marking endogenous dsRNAs as “self”, thereby preventing unintended activation of host PRRs while allowing accurate immune responses to foreign dsRNAs. This regulatory function is essential because dsRNAs-acting as PAMPs-are commonly produced or accumulated during most viral infections, either as the viral genome in dsRNA viruses or as replication intermediates in dsDNA, ssDNA and ssRNA viruses within host cells (52–54). By editing self-derived dsRNAs, ADAR1 ensures proper discrimination between self and non-self RNA, thereby supporting effective antiviral defense.

PRRs that detect dsRNAs can be broadly classified into three main categories based on their downstream signaling pathways (53). The first category includes RIG-I-like receptors (RLRs, e.g., RIG-I and MDA5) and TLR3, which initiate IFN-β production upon viral dsRNA recognition via mitochondrial antiviral signaling protein (MAVS) and Toll/interleukin-1 receptor (TIR)-domain-containing adapter-inducing interferon-β (TRIF), respectively. This activates IFN signaling and induces hundreds of interferon-stimulated genes (ISGs), forming the first line of innate antiviral defense (53). The second category consists of PKR and 2’-5’-oligoadenylate synthase (OAS), which primarily inhibit cell growth. PKR binds dsRNAs, undergoes dimerization and autophosphorylation, and phosphorylates the translation initiation factor eIF2α, leading to global translational arrest. Similarly, OAS proteins are activated by dsRNAs and synthesize 2’-5’-phosphodiester-linked oligoadenylates (2’-5’A), which activate latent endoribonuclease L (RNase L). Activated RNase L degrades viral RNA and most cytosolic RNAs-including rRNA, tRNA, and mRNA-thereby suppressing translation and affecting cell growth and differentiation (53, 55). The third category is the nucleotide-binding oligomerization domain (NOD)-, leucine-rich repeat (LRR)-, and pyrin domain-containing 1 (NLRP1) inflammasome, which senses dsRNAs via its leucine-rich repeat domain (53, 56, 57). Ligand recognition induces inflammasome assembly and caspase-1 activation. Caspase-1 cleaves the pore-forming protein, gasdermin D (GSDMD) to form membrane pores that execute pyroptosis and processes pro-IL-1β and pro-IL-18 into mature inflammatory cytokines (53, 56). Together, these dsRNA-sensing pathways underscore the importance of distinguishing self from non-self dsRNAs. Misidentification of endogenous dsRNAs can lead to severe outcomes, such as type I interferonopathies, cytokine storms, and aberrant cell death.

### 2.3 Consequences of ADAR1 loss: activation of dsRNA sensors

Loss of ADAR1 function-and the resulting accumulation of unedited self-dsRNAs-leads to aberrant activation of dsRNA-sensing pathways. Deletion of the cytosolic isoform ADAR1p150 activates both MDA5 and PKR in humans and mice. Notably, embryonic lethality and shortened lifespan observed in *Adarp150*^-/-^ mice are completely rescued by concurrent deletion of MDA5 and PKR, demonstrating that ADAR1 is indispensable for maintaining immune homeostasis by suppressing these pathways (58). A similar requirement for ADAR1 has been reported in neuronal progenitor cells, where ADAR1 knockout induces spontaneous IFN production, PKR activation, and cell death (59). Furthermore, introducing a PKR mutation in ADAR1/MAVS double-mutant mice rescues all pathological defects, prevents lethality, and enables long-term survival (60). Together, these findings illustrate that ADAR1 finely balances antiviral defense and immune tolerance by preventing inappropriate activation of PKR- and MDA5-dependent signaling through its A-to-I RNA editing activity. Consistently, a recent study showed that ADAR1-mediated A-to-I editing reduces the MDA5-dependent immunogenicity of endogenous long dsRNAs, further supporting the critical role of the ADAR1-dsRNAs-MDA5 axis in maintaining host immune homeostasis (61). Indeed, chronic activation of MDA5 and IFN signaling resulting from ADAR1 deficiency leads to severe autoinflammatory phenotypes (62).

Beyond MDA5 and PKR, ADAR1 also limits cytosolic RNA sensing by RIG-I through a deamination-independent mechanism (63). In HEK293 cells, endogenous RNAs trigger type I IFN production in the absence of ADAR1, and inducible ADAR1 knockout mice demonstrate excessive IFN induction in neuronal tissues. These findings indicate that ADAR1 directly prevents aberrant recognition of self dsRNAs by RIG-I (63).

Similarly, ADAR1 constrains the OAS–RNase L pathway. ADAR1 deletion in human A549 lung epithelial cells results in a lethal phenotype that can be rescued by CRISPR/Cas9-mediated RNase L knockout, pharmacological RNase L inhibition, or expression of the viral RNase L antagonist NS2 (64, 65). Interestingly, RNase L ablation improves cell survival even in the presence of MDA5 and MAVS, suggesting that OAS-RNase L may serve as a primary self-dsRNAs-responsive pathway leading to cell death under ADAR1 deficiency (64).

The role of ADAR1 in regulating the nucleotide-binding domain of leucine-rich repeat protein 1 (NLRP1) inflammasome remains unclear. However, a related study showed that ADAR1 overexpression impairs NLRP3 inflammasome activation in LPS + palmitic acid-treated THP-1 cells, whereas ADAR1 deficiency enhances NLRP3 activation (66). These findings raise the possibility that ADAR1 may also attenuate NLRP1-driven inflammation and pyroptosis, though this requires further investigation.

Collectively, these findings highlight that ADAR1 is essential for immune homeostasis. By editing and masking endogenous dsRNAs, ADAR1 prevents aberrant activation of host dsRNA sensors-including PKR, RLRs, and the OAS-RNase L pathway-thereby safeguarding against self-RNA-induced autoinflammation while preserving antiviral immunity.

## 3 ADAR1 modulates the battles between host and non-self-virus

**Figure 2:**
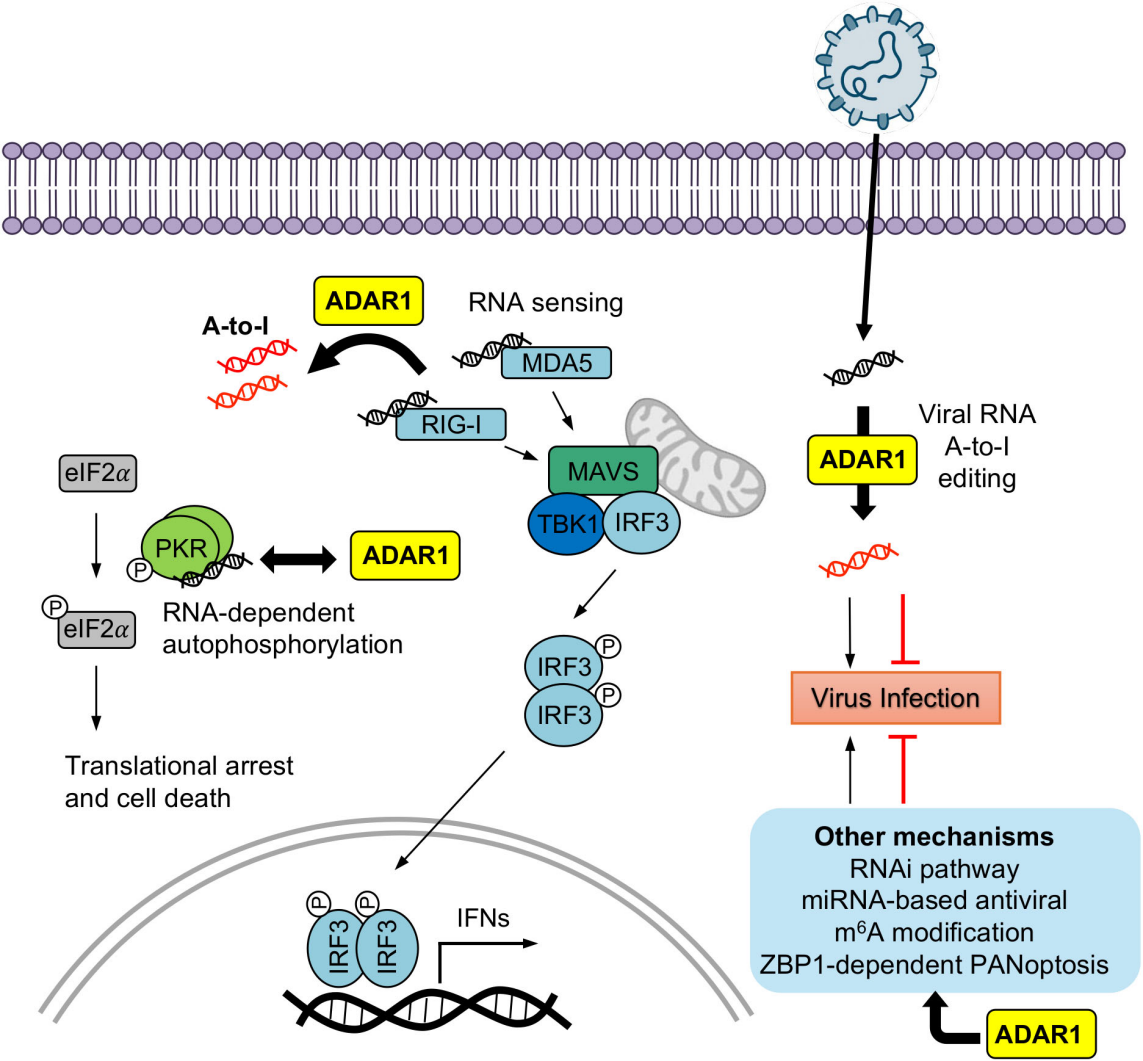
ADAR1 modulates host-virus interactions through RNA sensing and regulatory pathways. ADAR1 catalyzes A-to-I editing of dsRNAs to regulate antiviral responses and viral replication. (1) By editing endogenous dsRNAs, ADAR1 suppresses activation of the RIG-I and MDA5 pathways, thereby limiting MAVS-TBK1-IRF3 signaling and type I IFN production. In parallel, ADAR1 directly interacts with and restrains PKR activation, reducing PKR-mediated eIF2α phosphorylation, translational arrest, and cell death. Many viruses exploit these ADAR1-dependent immune-suppressive functions to promote infection, whereas for some viruses ADAR1-PKR regulation can restrict replication. (2) In addition, ADAR1 edits viral RNAs, introducing A-to-I substitutions that can have either proviral (black arrow) or antiviral (red arrow) effects depending on the viral species and editing sites. (3) ADAR1 also interfaces with other antiviral mechanisms, including RNA interference (RNAi), miRNA-mediated regulation, m^6^A RNA modification, and ZBP1-dependent PANoptosis.

ADAR1-mediated A-to-I editing occurs in both self and viral dsRNAs (34, 67, 68). It acts as both proviral and antiviral effectors not only via modulating PRR activation and editing viral genomes, but also through cooperating with other RNA modifications, such as m^6^A system (**Figure 2**, **Table 1**). Moreover, evidence about ADAR1 regulation of apoptosis or other antiviral programs, including miRNA-based antiviral defense, to modulate virus infection has also been revealed (**Figure 2**, **Table 1**).

**Table 1:** Proviral and antiviral activities of ADAR1 during viral infections.

ADAR1-regulated mechanisms	Proviral	Antiviral
ADAR1-PKR axis	Increased ADAR1p150 expression and decreased PKR activation [Coronavirus (69), HIV-1 (72)]	PKR selectively suppresses 5’-cap-dependent translation [HCV (95)]
	Viral infection triggers the association of ADAR1 and PKR [HSV (70)]	ADAR1 reverses circRNAs-mediated suppression of PKR by downregulating its expression [EMCV (98)]
	ADAR1-PKR signaling favors viral infection [VSV (71), MeV (74, 75), ORFV (76), HTLV1/2 (77)]	
	ADAR1 Inhibits ZIKV-induced PKR activation [ZIKV (73)]	
ADAR1-RLR axis	ADAR1 reduces RIG-I RNA binding [SeV (63)]	
	ADAR1 inhibits RLR-dependent IFN expression [IAV (79), KSHV (84)]	
	Increased ADAR1p150 reduces RIG/MDA5 recognition of edited HBV RNA [HBV (81)]	
	ADAR1 edits MAVS to decrease RIG/MDA5 signaling [HBV (82)]	
	ADAR1 decreases RIG/MDA5 expression [HIV (83)]	
ADAR1-mediated A-to-I editing	Hyper-editing of the amber stop codon (UAG) [HDV (85, 86)]	A-to-I editing in S gene [SARS-CoV-2 (99)]
	HIV RNA genome, including 5’ UTR and the Rev and Tat coding sequences [HIV (87)]	Hyper-editing at non-amber/W sites within the viral genome [HDV (100)]
	Viral genomes [MeV (75), SARS-CoV-2 (89)]	Editing of the *rev* and RRE regions within the *env* gene [HIV (102)]
ADAR1 crosstalk with other mechanisms	RNAi pathway [SeV (91)]	Increase miRNA-122 expression and processing miR-122 [HBV (103)]
	miRNA-based antiviral defenses [DENV (92)]	Inhibit ZBP1-dependent PANoptosis [SARS-CoV-2 (104)]
	Adaptive immunity [SARS-CoV-2 (35)]	
	m^6^A modification [VSV (93)]	
	Unknown mechanisms [CHIKV/VEEV (94)]	

### 3.1 ADAR1 pro-viral activities

ADAR1 edits A-to-I in self-dsRNAs to prevent recognition by host PRRs. As a gatekeeper of the RNA-sensing pathway, ADAR1 primarily supports viral infection by suppressing PRR activation, thereby limiting type I IFN responses. In addition, its RNA-editing activity introduces A-to-I mutations into viral genomes or viral gene transcripts, further contributing to viral replication and evolution. Remarkably, emerging studies have revealed that ADAR1 also targets other antiviral pathways, including the RNA interference (RNAi) machinery, to facilitate viral infection. Collectively, ADAR1 promotes viral replication through multiple, distinct mechanisms.

#### 3.1.1 ADAR1 promotes viral infection by suppressing PKR/RLR recognition of non-self-viral dsRNA

Although ADAR1 protects the host by distinguishing self from non-self to maintain immune homeostasis, growing evidence indicates that ADAR1 is frequently hijacked by viruses to evade immune detection and create a favorable environment for replication. Acting as an immune balancer, ADAR1 modulates the activation of three major RNA sensors-PKR, RIG-I, and MDA5-to prevent inappropriate immune activation. However, many viruses have evolved to exploit these regulatory mechanisms for their own benefit.

PKR-mediated antiviral defense is a critical translational checkpoint that restricts viral infection. Through co-evolution, multiple viruses have adopted ADAR1-dependent strategies to evade PKR-mediated immune restriction. For example, coronaviruses utilize their spike (S) protein-which exhibits high mutation rates linked to enhanced virulence and adaptability-to upregulate ADAR1p150 expression via the transcription factor TCF7L2. This leads to ribonuclease-mediated cleavage of edited dsRNAs, reduced intracellular viral dsRNA levels, and inhibition of the PKR-eIF2α pathway, translation arrest, and stress granule (SG) formation, collectively facilitating viral infection (69). Similarly, herpes simplex virus 1 (HSV-1) exploits ADAR1p150, which associates with PKR to suppress PKR/eIF2α-mediated translational arrest, likely through competitive binding and/or dsRNA editing (70). ADAR1 also enhances host susceptibility to vesicular stomatitis virus (VSV) by interacting with PKR and inhibiting its kinase activity in an RNA-editing-independent manner (71).

In the case of human immunodeficiency virus type 1 (HIV-1), ADAR1p150 expression is upregulated, and the virus employs the Z-DNA binding motif and dsRNA binding domains-but not the catalytic domain-of ADAR1p150 to suppress PKR activation and promote viral replication (72). Zika virus (ZIKV) also co-opts ADAR1 to impair PKR activation and enhance viral protein translation via an RNA-editing-independent mechanism (73). Likewise, measles virus (MeV) selectively exploits ADAR1’s ability to inhibit dsRNA-dependent antiviral responses mediated by PKR and interferon regulatory transcription factor 3 (IRF3), thereby promoting viral growth and suppressing virus-induced apoptosis (74, 75). ADAR1-mediated inhibition of PKR phosphorylation has also been shown to facilitate infection by Orf virus (ORFV) (76) and human T-cell leukemia virus type 1 and 2 (HTLV-1/2) (77).

Despite these findings, the precise mechanisms of ADAR1 antagonism against PKR remain incompletely defined and may involve dsRNA destabilization, competition, or sequestration (78). Targeting the counteractions between ADAR1 and PKR may thus represent a promising strategy to control viral infections. Beyond PKR regulation, viruses also exploit ADAR1 to evade RIG-I-mediated sensing. ADAR1 limits RIG-I recognition of viral RNA by reducing RIG-I binding through its RNA-binding, but not RNA-editing activity (63). Preventing sustained RIG-I-induced IFN-β expression and apoptosis is another ADAR1-dependent mechanism that supports influenza A virus (IAV) infection (79). MDA5, another RLR family member, recognizes long viral dsRNAs to activate type I IFN responses. ADAR1 is a well-established negative regulator of MDA5, preventing overactivation of innate immunity under steady-state conditions. Although the precise role of the ADAR1-MDA5 axis during viral infection is not fully elucidated, several studies suggest a proviral effect. ADAR1 deficiency disrupts RNA editing and aberrantly activates MDA5, leading to type I interferonopathies (80), while loss of ADAR1-mediated RNA editing specifically triggers MDA5 activation (58).

A clear example of the proviral role of ADAR1-MDA5 regulation is observed during hepatitis B virus (HBV) infection. The HBV X protein (HBx) transcriptionally enhances ADAR1 expression, particularly the cytoplasmic p150 isoform, which subsequently edits HBV RNA at specific adenosine residues, generating A-to-I substitutions that alter viral RNA secondary structure and protein-coding potential. These editing events reduce the recognition of HBV RNA by RIG-I and MDA5, thereby suppressing type I IFN induction and facilitating viral replication (81). Additionally, ADAR1 may facilitate HBV infection by interfering with RIG-I/MDA5 signaling through downregulation of MAVS. This suppression of MAVS expression is attributed to ADAR1-mediated A-to-I editing in the 3’UTR of MAVS mRNA, including the chr20:3870562 site, which alters mRNA stability via human antigen R (HuR)-dependent post-transcriptional regulation (82). Consistent with these findings, ADAR1 knockdown in primary macrophages enhances RIG-I, MDA5, and IFN expression, conferring resistance to HIV infection and supporting a proviral role of ADAR1 through negative regulation of RLR-mediated sensing (83). Similarly, Kaposi’s sarcoma-associated herpesvirus (KSHV) exploits ADAR1-mediated suppression of RLR/IFN axis; ADAR1 deficiency markedly increases RLR-dependent IFN production, which inhibits KSHV lytic replication (84). Collectively, these studies indicate that the ADAR1-PKR/RIG-I/MDA5 axis, which normally maintains immune homeostasis, can be subverted by diverse viruses to promote productive infection.

#### 3.1.2 ADAR1-mediated viral RNA editing supports viral infection

Apart from enabling viruses to evade dsRNA sensors and escape host immune surveillance, many viruses exploit the RNA-editing activity of ADAR1 to enhance replication and adaptability through targeted mutations in viral genomes or protein-coding sequences. ADAR1-mediated A-to-I editing plays an essential role in hepatitis delta virus (HDV) infection (85). Hyper-editing of the amber stop codon (UAG) which normally terminates synthesis of hepatitis delta antigen (HDAg-S), leads to overproduction of the long isoform (HDAg-L), a key factor for efficient viral replication (85, 86). In HIV infection, ADAR1 not only stimulates viral replication through editing-independent mechanisms but also promotes viral survival via its editing-dependent activity. By binding HIV-1 transcripts, ADAR1 induces A-to-G mutations in viral sequences, including the 5’ UTR and the Rev and Tat coding regions, which enhances viral infectivity by facilitating virion release and infection (87). Furthermore, A-to-I editing of HIV RNA contributes to increased viral diversity and adaptability during infection (85, 87, 88). Similarly, the editions elicited by ADAR1 on MeV genomes are required for efficient viral replication (75). ADAR1-mediated RNA editing also occurs during SARS-CoV-2 infection, where a conserved long-range RNA interaction recruits ADAR1 to specific viral regions, increasing editing frequency and thereby enhancing viral fitness (89). Consistently, a strong correlation between COVID-19 disease and elevated A-to-I RNA editing activity in the host has been observed (90). Collectively, these findings highlight that ADAR1-mediated RNA editing not only contributes to immune evasion but also directly promotes viral replication and evolution across diverse viral families.

#### 3.1.3 Other proviral activities of ADAR1

Beyond its role in masking viral dsRNAs and promoting RNA editing-mediated mutations, ADAR1 also interferes with other antiviral programs to facilitate viral replication. One of its key targets is the RNAi pathway. ADAR1-mediated editing can disrupt microRNA (miRNA)-silencing motifs, thereby diminishing RNAi effectiveness and enabling viral escape, as observed in the case of Sendai virus (91). Similarly, miRNA-3614-5p suppresses dengue virus (DENV) infection by downregulating the proviral ADAR1 expression. Overexpression of miR-3614-5p reduces DENV replication in wild-type (WT) mouse embryonic fibroblasts (MEFs) but not in ADAR knockout (KO) cells, indicating that ADAR1 impairs miRNA-based antiviral defenses to favor DENV replication (92).

In addition, ADAR1 modulates host immunity through antigen-presenting cells (APCs). The ablation of ADAR1 in APCs enhances the presence of inflammatory conventional dendritic cells type 2, promotes lung infiltration of activated tissue-resident memory T cells, and increases resistance to early SARS-CoV-2 infection. These findings suggest that ADAR1 exerts a proviral role by fine-tuning the antiviral state of APCs, thereby influencing downstream adaptive immune activation (35).

Notably, ADAR1 also cooperates with other RNA modification pathways to suppress innate antiviral signaling. For example, N6-methyladenosine (m^6^A) modification can augment ADAR1 activity: the m^6^A reader protein YTH N^6^-methyladenosine RNA binding protein 1 (YTHDF1), which binds m^6^A-modified transcripts and promotes translation, upregulates ADAR1p150 expression and enhances A-to-I RNA editing in response to IFN stimulation. This interaction suppresses antiviral IFN signaling and promotes viral replication (93).

Furthermore, ADAR1 overexpression enhances the replication of chikungunya virus (CHIKV) and Venezuelan equine encephalitis virus (VEEV), although the underlying mechanism remain to be elucidate (94). Together, these findings demonstrate that ADAR1 functions through multiple mechanisms-targeting RNAi, modulating immune cell activation, and cooperating with RNA modification pathways-to broadly support viral infection.

### 3.2 ADAR1 antiviral activities

Although numerous studies have highlighted the proviral functions of ADAR1-such as suppressing PKR activation and editing viral RNAs to enhance viral replication-accumulating evidence also indicates that ADAR1 can function as an antiviral factor under specific contexts. ADAR1 exerts antiviral activity by modulating PKR activation, performing inhibitory RNA editing on viral genomes, and supporting additional antiviral programs such as miRNA-mediated regulation and the control of cell survival pathways. Collectively, these findings underscore the dual nature of ADAR1 as both a host factor and a viral collaborator, depending on the infection context and cellular environment.

#### 3.2.1 ADAR1 modulates PKR activation to inhibit viral infection

PKR is a well-established dsRNA sensor that restricts viral infection by phosphorylating eIF2α, leading to host translational arrest. While ADAR1 often suppresses PKR activation to favor viral replication, in certain viral contexts it can instead contribute to antiviral outcomes.

During hepatitis C virus (HCV) infection, PKR activation paradoxically promotes viral replication by selectively inhibiting ISG expression (95) and blocking 5′-cap-dependent translation, while leaving internal ribosome entry site (IRES)-dependent translation initiation of HCV RNA unaffected (67, 95). This selective translational advantage allows HCV to maintain viral protein synthesis under host stress conditions. Interestingly, ADAR1 enhances IFN sensitivity in HCV replicon systems (96), suggesting that its suppression of PKR may in fact counteract the proviral effect of PKR activation, thereby functioning in an antiviral capacity against HCV.

A similar antiviral mechanism may operate in encephalomyocarditis virus (EMCV) infection. EMCV generates viral circular RNAs (circRNAs) that contain 16–26 bp imperfect RNA duplexes, which antagonize PKR activation and promote viral replication (67, 97). ADAR1, however, can downregulate the expression of circRNAs (98), potentially enhancing PKR activation and thereby restricting EMCV replication. These findings collectively indicate that ADAR1 can exert antiviral effects by fine-tuning PKR activity to restore proper antiviral signaling.

#### 3.2.2 ADAR1-mediated RNA editing inhibits viral infection

In addition to modulating PKR signaling, ADAR1 can inhibit viral replication through its RNA-editing activity, depending on the location and consequence of A-to-I (A→G) mutations. In SARS-CoV-2, A→G substitutions within the S gene-potentially catalyzed by ADAR1-are found more frequently in clinical samples with lower viral loads. This correlation implies that ADAR1-mediated RNA editing may compromise viral fitness and infectivity, acting as a host restriction mechanism (99). Similarly, ADAR1 restricts HDV replication through extensive hyper-editing at non-amber/W sites within the viral genome. These editing events generate variant forms of the HDAg that function as trans-dominant inhibitors of HDV RNA replication, thereby limiting viral propagation (100).

ADAR1 also exhibits antiviral functions during HIV-1 infection. The enzyme contributes to A→G hypermutation patterns observed in HIV-1-infected lung tissues, and *in vitro* studies confirm that ADAR1 suppresses viral replication post-transcriptionally in infected macrophages (101). Mechanistically, ADAR1 editing of the *rev* and *RRE* regions within the *env* gene impairs nuclear export of *gag*, *pol*, and *env* mRNAs, leading to reduced viral protein synthesis and infectivity (102). Thus, depending on the editing sites and cellular context, ADAR1’s catalytic activity can effectively dampen viral replication rather than enhance it.

#### 3.2.3 Other antiviral activities of ADAR1

Beyond regulating dsRNA sensors and viral RNA editing, ADAR1 also contributes to antiviral defense through modulating host signaling pathways and supporting cellular antiviral programs. One example involves microRNA-122 (miR-122), a liver-enriched miRNA known for its antiviral function against HBV. ADAR1 enhances both the expression and processing of miR-122, thereby reducing HBV replication (103). This indicates that ADAR1 can amplify antiviral miRNA responses to limit viral persistence in hepatocytes.

ADAR1 may also protect host cells by preventing pathological cell death during viral infection. During SARS-CoV-2 infection, activation of cyclic GMP-AMP synthase (cGAS)-stimulator of interferon genes (STING) pathway induces autophagic degradation of ADAR1, leading to the accumulation of Z-nucleic acids and triggering Z-DNA binding protein 1 (ZBP1)-dependent PANoptosis, a form of inflammatory programmed cell death. The loss of ADAR1 thereby enhances tissue damage and inflammatory responses. This observation suggests that ADAR1 functions as a negative regulator of ZBP1-dependent PANoptosis, helping to limit SARS-CoV-2-induced pathology (104). Together, these findings highlight that ADAR1’s antiviral roles extend beyond direct RNA editing. By enhancing antiviral miRNA activity and suppressing pro-inflammatory cell death pathways, ADAR1 acts as a multifaceted regulator of host defense mechanisms.

### 3.3 Viral regulation of ADAR1 activity

ADAR1 plays a critical role in antiviral defense by modulating the activation of dsRNA sensors, introducing A-to-I editing on viral genomes, and engaging apoptosis and other antiviral pathways, including miRNA-based defenses. However, as obligate intracellular parasites that rely entirely on host cells for replication (105), viruses have also evolved strategies to manipulate ADAR1 activity during an ongoing host-virus arms race. One clear example is seen with vaccinia virus (VV). The VV-encoded RNA binding protein E3L-an essential IFN-resistance factor-shares structural features with ADAR1, including Z-DNA and dsRNA-binding domains that recognize Z-form nucleic acids or dsRNAs. E3L potently inhibits ADAR1 deaminase activity through both its C-terminal and N-terminal domains (106). Interestingly, similar to ADAR1, E3L also antagonizes IFN-inducible antiviral pathways mediated by PKR and OAS (106, 107), contributing to IFN resistance and enhanced viral replication. These findings suggest that the E3L inhibition of ADAR1 is independent of PKR and OAS signaling, implying that ADAR1 may restrict VV infection primarily through editing of the viral genome. Another example is the adenovirus-associated (VAI) RNA, which also suppresses ADAR1-mediated RNA editing (108). In contrast, DENV NS3 and IAV NS1 proteins enhance ADAR1 editing activity to promote viral replication, highlighting the context-dependent regulation of ADAR1 by viruses (109). Although current evidence clearly shows that viruses can modulate the A-to-I editing activity of ADAR1, our understanding of how viral factors specifically influence ADAR1’s catalytic deamination mechanism remains limited. Further studies are needed to elucidate the molecular mechanisms by which viruses alter ADAR1 function during infection.

## 4 Concluding remarks

ADAR1 is a pivotal regulator of RNA sensing during viral infection, but its functions extend far beyond preventing PRR overactivation. Many viruses actively exploit ADAR1-through modulation of its expression or editing activity-to suppress antiviral signaling and promote replication. Conversely, ADAR1 can also exert antiviral effects by editing viral genomes, modulating PKR activity, or influencing infection-induced cell death. These dual roles highlight ADAR1 as a switch that fine-tunes the balance between antiviral defense and viral survival. Recent findings suggest that ADAR1 intersects with other RNA regulatory pathways, including RNA interference, m^6^A-mediated modification, and PANoptosis-mediated forms of cell death (48, 74, 91, 93, 110–112). These interactions may determine infection outcomes in a virus-specific and context-dependent manner-potentially shaping viral evolution, tissue tropism, and disease severity. The complexity of ADAR1 function suggests that therapeutic strategies must move beyond simple inhibition. Selectively targeting ADAR1 isoforms, editing sites, or interaction partners may allow modulation of its proviral functions while preserving its essential role in immune homeostasis. As our understanding of RNA modification networks advances, ADAR1 stands at a promising crossroads for developing host-directed antiviral therapies.
